# Clinical Features and Comparison of *Kingella* and Non–*Kingella* Endocarditis in Children, Israel

**DOI:** 10.3201/eid2703.203022

**Published:** 2021-03

**Authors:** Alexander Lowenthal, Hila Weisblum-Neuman, Einat Birk, Liat Ashkenazi-Hoffnung, Itzhak Levy, Haim Ben-Zvi, Gabriel Amir, Georgy Frenkel, Elchanan Bruckheimer, Havatzelet Yarden-Bilavsky, Dafna Marom, Eran Shostak, Elhanan Nahum, Tamir Dagan, Gabriel Chodick, Oded Scheuerman

**Affiliations:** Tel-Aviv University, Tel-Aviv, Israel (A. Lowenthal, H. Weisblum-Neuman, E. Birk, L. Ashkenazi-Hoffnung, I. Levy, H. Ben-Zvi, G. Amir, G. Frenkel, E. Bruckheimer, H. Yarden-Bilavsky, D. Marom, E. Shostak, E. Nahum, T. Dagan, G. Chodick, O. Scheuerman);; Schneider Children’s Medical Center of Israel, Petach-Tikva, Israel (A. Lowenthal, H. Weisblum-Neuman, E. Birk, L. Ashkenazi-Hoffnung, I. Levy, G. Amir, G. Frenkel, E. Bruckheimer, H. Yarden-Bilavsky, E. Shostak, E. Nahum, T. Dagan, O. Scheuerman);; Rabin Medical Center, Beilinson Hospital, Petach-Tikva (H. Ben-Zvi);; Tel Aviv Sourasky Medical Center, Tel Aviv (D. Marom)

**Keywords:** *Kingella* species, *Staphylococcus aureus*, *Streptococcus* species, infective endocarditis, oral aphthae, Israel, Streptococci, bacteria, bacterial infection, pediatric, bacteremia

## Abstract

*Kingella* spp. have emerged as an important cause of invasive pediatric diseases. Data on *Kingella* infective endocarditis (KIE) in children are scarce. We compared the clinical features of pediatric KIE cases with those of *Streptococcus* species IE (StIE) and *Staphylococcus aureus* IE (SaIE). A total of 60 patients were included in the study. Throughout the study period, a rise in incidence of KIE was noted. KIE patients were significantly younger than those with StIE and SaIE, were predominately boys, and had higher temperature at admission, history of oral aphthae before IE diagnosis, and higher lymphocyte count (p<0.05). Pediatric KIE exhibits unique features compared with StIE and SaIE. Therefore, in young healthy children <36 months of age, especially boys, with or without a congenital heart defect, with a recent history of oral aphthae, and experiencing signs and symptoms compatible with endocarditis, *Kingella* should be suspected as the causative pathogen.

Infective endocarditis (IE) is a rare but potentially life-threatening disease in children and has an incidence of 0.8–3.3 cases/1,000 pediatric hospital admissions (*1*). Although early reports described IE exclusively in children whose hearts were structurally abnormal because of congenital heart disease or acquired rheumatic heart disease, this infection has more recently been reported in diverse groups of patients. In addition to children with congenital heart disease, other groups of children have emerged as being at high risk for IE, including children born prematurely; those with noncardiac congenital malformations, genetic syndromes, and malignancies; and, in particular, children with central venous catheters and those who have been treated by invasive procedures or intravenous medications (*1*–*3*).

The most common IE pathogens in children are gram-positive cocci, especially the α-hemolytic viridans group streptococci (e.g., *Streptococcus sanguis, S. mitis* group, and *S. mutans*), staphylococci, and enterococci. In patients with IE who are >1 year of age, the viridans group streptococci are the most commonly isolated organisms. *Staphylococcus aureus* is the second most common cause of IE in children but the most common cause of acute bacterial endocarditis (*2*). The HACEK group (*Haemophilus parainfluenzae, H. aphrophilus, H. paraphrophilus, Aggregatibacter actinomycetemcomitans, Cardiobacterium hominis, Eikenella* spp., and *Kingella kingae*) is a rare cause of IE, accounting for ≈1.4% of all cases of endocarditis (*2*,*4*).

*Kingella* spp. are carried asymptomatically in the oropharynx and disseminate through close interpersonal contact. These gram-negative bacteria (especially *K. kingae*) are commonly the etiology of pediatric bacteremia and the leading cause of osteomyelitis and septic arthritis in children 6–36 months of age (*5*). Invasive *K. kingae* disease usually affects previously healthy children <4 years of age, whereas older children and adults frequently have predisposing conditions (*6*).

*Kingella* IE (KIE) is estimated to account for 0%–6% of all IE cases in the general population (*7*–*10*). Similar numbers have been described in the pediatric population in a few published reports. *Kingella* appears to cause an even higher number of endocardial infections in children and was the etiologic agent of 4 (7.8%) of 51 episodes in a tertiary-care pediatric hospital in Israel (*3*) and of 6 (7.1%) of 85 cases among New Zealand children (*11*). However, lower rates of KIE have also been reported; a recent study of 53 cases of IE in Belgium described no cases of KIE (*12*). Serious cardiovascular and central nervous system complications and a need for emergent cardiac surgery for life-threatening complications that do not respond to conservative medical treatment have been described in the pediatric population (*13*). *Kingella* spp. as a causative pathogen of endocarditis has been poorly studied, and the number of studies regarding the pediatric population is limited (*3*,*11*,*14*–*20*). Therefore, we examined the characteristics of pediatric KIE case-patients to compare these cases with IE cases caused by other common pathogens.

## Methods

We retrospectively reviewed all files of children with IE admitted to Schneider Children’s Medical Center of Israel (Petach-Tikva, Israel) during 1994–2019. We included children <18 years of age with history and physical findings consistent with possible or definite diagnosis of IE according to the Duke criteria (*21*). Culture-negative IE cases were excluded because some might represent undiagnosed KIE. We also excluded cases of endocarditis that were attributed to coagulase-negative staphylococci species and other rare enteric gram-negative bacteria, because these consist of only nosocomial cases or IE cases associated with foreign bodies or intravenous catheters, which are epidemiologically distinct from the general IE pediatric population. A pediatric cardiologist and a pediatric infectious diseases specialist reviewed all files. Cases were divided into 2 groups on the basis of bacterial etiology: KIE (*K. kingae* and *K. dentrificans*) and non-*Kingella* IE (non-KIE, including *Streptococcus* species and *S. aureus*).

Each isolate was identified by using the VITEK 2 system (bioMérieux, https://www.biomerieux.com) or MALDI Biotyper System (Bruker, https://www.bruker.com), in accordance with the manufacturers’ instructions for bacteria identification. Antimicrobial-susceptibility profiles of the isolates were determined by the disk diffusion method (Oxoid, http://www.oxoid.com), Etest (bioMérieux), or VITEK 2 as needed and interpreted based on the Clinical and Laboratory Standards Institute criteria for other non-*Enterobacteriaceae* (*22*). Data retrieved from patients’ charts included demographics, past medical history, clinical manifestations, laboratory findings, imaging studies, treatment, and outcome. The characteristics of KIE were compared with characteristics of *Streptococcus* species IE (StIE) and *S. aureus* IE (SaIE). The study was approved by the local institutional review board.

### Statistical Analysis

To compare baseline correlates between case categories, we employed a χ^2^ test for categorical variables, analysis of variance test for parametric continuous data, and Kruskal–Wallis test for nonparametric continuous data. We calculated p values for the post hoc comparison with Bonferroni correction for the number of comparisons. Statistical analyses were performed by using SPSS Statistics 23.0 software (IBM, https://www.ibm.com) and the tableone package (*23*) in R version 3.6.1 (R Foundation for Statistical Computing, https://www.r-project.org).

## Results

### Study Population

During the study period, IE was diagnosed in 114 admitted patients, yielding an incidence rate of 1.4 cases/1,000 admissions. A total of 60 patients with IE caused by *Kingella* species, *Streptococcus* species, or *S. aureus* were included in this study. In 19 patients (14% of total IE admissions), the causative pathogen was *Kingella* species (*K. kingae* [n = 18] and *K. dentrificans* [n = 1]); in 25 patients (19%), the causative pathogen was *Streptococcus* species (*S. viridans* [n = 17], *S. pneumoniae* [n = 6], and *S. pyogenes* [n = 2]); and in 16 patients (12%), the causative pathogen was *S. aureus*.

### Baseline Characteristics

The baseline characteristics of study participants with KIE and non-KIE are detailed in Table 1. Patients with KIE were significantly younger than those with non-KIE (16 + SD 10.29 months vs. 91 + SD 74.11 months; p<0.001). Although the difference was not statistically significant, congenital heart disease was previously diagnosed in fewer patients with KIE than in patients with non-KIE (53% vs. 78%; p = 0.09). Based on queries regarding a previous heart murmur, far fewer patients with KIE had a history of a known murmur than those with non-KIE (37% vs. 71%; p = 0.027). All KIE cases were community-acquired. No statistically significant differences were observed in previous noncardiac disease and previous interventions (surgery, cardiac catheterization, and dental procedures) between the groups. Median time (weeks) between prior cardiac catheterization to infection was shorter for the non-KIE group than the KIE group but was not statistically significant.

**Table 1:** Comparison of baseline demographics and characteristics of pediatric infective endocarditis case-patients by causative pathogen, Israel, 1994–2019*

Characteristic	*Kingella,* n = 19 (32%)	Non*-Kingella,* n = 41 (68%)	p value
Age, mo, mean (SD)	16 (+ 10.29)	91.4 (+ 74.11)	<0.001
Sex			
F	6 (32)	25 (61)	0.065
M	13 (68)	16 (39)	
Congenital heart disease	10 (52)	32 (78.0)	0.09
Known heart murmur	7 (37)	29 (71)	0.027
Recent dental procedure†	0 (0)	6 (15)	0.195
Long-term CVL	0 (0)	5 (12)	0.277
Recent catheterization†	4 (21)	6 (15)	0.804
Time from catheterization to infection, wk	14.50 (10–23)‡	6.00 (2.9–14)‡	0.24
Community-acquired infection	19 (100)	33 (80)	0.097

*Values are no. (%) except as indicated. CVL, central venous line. †Recent catheterization or dental procedures defined as <6 mo before diagnosis of infective endocarditis. ‡Median (interquartile range).

### Clinical, Laboratory, and Imaging Characteristics

We compiled the clinical, laboratory, and imaging characteristics of case-patients with KIE compared with non-KIE case-patients (Table 2). Patients with KIE had significantly higher fever when first examined (40°C [range 39.45°C–40°C] vs. 39°C [range 38.6°C–39.8°C]; p = 0.003). No difference was observed in duration of febrile disease before admission. Hepatosplenomegaly was more common among non-KIE patients. Approximately a quarter of KIE patients reported previous oral aphthae, significantly more than those in the non-HIE group (5 patients vs. 0 patients; p = 0.003). No additional differences in clinical findings were noted.

**Table 2:** Comparison of clinical, laboratory, and imaging characteristics of pediatric infective endocarditis case-patients by causative pathogen, Israel, 1994–2019*

Characteristic	*Kingella,* n = 19 (32%)	Non-*Kingella,* n = 41 (68%)	p value
Temperature, °C, median (IQR)	40 (39.45–40)	39 (38.6–39.8)	0.003
Fever duration before admission, d, median (IQR)	7 (4.50–14)	6 (3–14)	0.43
Hepatosplenomegaly	4 (21)	24 (58)	0.015
Oral aphthae	5 (26)	0 (0)	0.003
Ocular findings	1 (5)	8 (19)	0.294
Systemic emboli	7 (37)	14 (34)	1
Pulmonary emboli	0 (0.0)	2 (5)	0.837
Seizures	3 (16)	6 (15)	1
New onset murmur	7 (37)	10 (24)	0.492
Conduction disturbance	1 (5)	2 (5)	1
Microhematuria	5 (26)	23 (56)	0.061
Leukocyte count K/mL, mean (SD)	20.87 (+ 12.39)	16.39 (+ 9.58)	0.131
Neutrophils K/mL, mean (SD)	12.98 (+ 8.49)	12.43 (+ 8.67)	0.821
Lymphocytes K/mL, mean (SD)	4.27 (+ 3.04)	2.21 (+ 1.81)	0.002
NLR, mean (SD)	4.7 (6.71)	10.7 (11.3)	0.055
Hemoglobin, mean (SD)	10.00 (+ 1.36)	10.39 (+ 2.27)	0.489
Platelets (100 K/mL), mean (SD)	220.16 (+ 203.30)	243.85 (+ 178.53)	0.649
C-reactive protein, mean (SD)	12.56 (+ 6.79)	12.49 (+ 10.75)	0.979
ESR, mean (SD)	65.92 (+ 38.86)	64.19 (+ 34.34)	0.89
No. positive blood cultures, median (IQR)	1 (1–2)	4 (3–5)	<0.001†
Time to eradication, d, median (IQR)	2 (2–3)	3 (2–5)	0.017
Echocardiography			
Mural thrombus	1 (5)	2 (5)	1
Reduced ventricular function	4 (21)	11 (27)	0.873
Vegetation	12 (63)	20 (49)	0.447
Left-sided involvement‡	12 (80)	15 (65)	0.297
Major Duke criteria			
Culture	7 (37)	40 (98)	<0.001
Echocardiography	16 (84)	23 (56)	0.067
Minor Duke criteria			
Fever	19 (100)	39 (95)	0.837
Congenital heart disease	10 (52)	31 (76)	0.138
Vascular	8 (42)	14 (34)	0.759
Immunologic	0 (0)	9 (22)	0.068
Blood culture	11 (58)	1 (2)	<0.001
Summary			
Definite	14 (74)	30 (73)	0.768
Possible	5 (23)	11 (27)	0.768

*Values are no. (%) except as indicated. All laboratory results are at admission apart from temperature, which we recorded as the highest in the 24 h before admission. ESR, erythrocyte sedimentation rate; IQR, interquartile range; NLR, neutrophil–lymphocyte ratio. †By Mann–Whitney test. ‡Percentage of left-sided involvement in patients with echocardiographic findings.

The leukocyte count at admission differed significantly in lymphocyte counts: 4.27 K cells/mL (3.04) among KIE case-patients vs. 2.21 K cells/mL (1.81) in non-KIE case-patients (p = 0.002). Study groups approached significance (p = 0.055) in neutrophil–lymphocyte ratio; patients with KIE had the lowest ratio (4.7), whereas non-KIE patients had a higher ratio (10.7). No differences were observed in other parameters of the complete blood count or the level of inflammatory markers between the 2 groups. The number of positive cultures differed significantly between the 2 groups; most patients with KIE had 1–2 positive blood cultures, and none had >4 positive cultures, compared with an average of 4 in the non-KIE group (p<0.001). Days to blood-culture sterilization were fewer in the KIE group (2 days [2–3] vs. 3 days [2–5]; p = 0.017). The chest radiography or echocardiography findings did not exhibit differential features between the 2 groups.

Duke criteria findings are listed in Table 2. Only 37% of those patients with KIE versus 98% in the non-KIE group (p<0.001) fulfilled the Duke major clinical criterion blood culture component. However, blood-culture positivity as a minor clinical criterion was far more prevalent in the KIE group than the non-KIE group (11 patients [58] vs. 1 patient [2]; p<0.001).

A post hoc comparison of the 3 pathogen groups (Table 3) showed that KIE differed significantly from the StIE group in a few parameters. Previous diagnosis of a heart murmur (p = 0.027) and hepatosplenomegaly (p<0.001) were less prevalent in patients with KIE. The absolute leukocyte count was significantly higher in the KIE group than in the StIE group (p<0.005). KIE was significantly more likely to be community-acquired than SaIE (p<0.012). Absolute neutrophil count was significantly lower in the KIE group than the SaIE group (p<0.001).

**Table 3:** Comparison of the characteristics of case-patients with infective endocarditis by specific causative pathogen, Israel, 1994–2019*

Characteristic	*Kingella species,* n = 19	*Streptococcus* species, n = 25	*Staphylococcus aureus* species, n = 16	p value
Age, mo, mean (SD)	16 (+ 10.29)	106.3 (+ 70.43)	68 (+ 75.89)	**<0.001**†
Sex				
F	6 (32)	15 (60)	10 (62)	0.104
M	13 (68)	10 (40)	6 (38)	
Congenital heart disease	10 (52)	21 (84)	11 (69)	0.079
Known murmur	7 (37)	19 (76)	10 (62.5)	**0.031**‡
Recent surgery	3 (16)	2 (8)	6 (37)	0.055
Recent dental procedure	0	5 (20)	1 (6.2)	0.077
Community-acquired infection	19 (100.0)	23 (92.0)	10 (62.5)	**0.003**§
Temperature, °C, median (IQR)	40 (39.45–40)	39 (39–39.6)	39 (38.4–40)	**0.013**†
Fever duration, d, median (IQR)	7 (4.5–14)	7 (2–21)	5 (3–7)	0.514
Hepatosplenomegaly	4 (21)	15 (60)	9 (56)	**0.025**‡
Oral aphthae	5 (26)	0	0	**0.002**†
Musculoskeletal infection	2 (11.8)	0	0	0.111
Microhematuria	5 (26.3)	13 (52.0)	10 (62.5)	0.08
Leukocyte count, K/mL, mean (SD)	20.87 (+ 12.39)	12.68 (+ 4.77)	22.18 (+ 12.23)	**0.005**‡
Neutrophils, K/mL, mean (SD)	12.98 (+ 8.49)	9.34 (+ 4.30)	17.27 (+ 11.41)	**0.012**§
Lymphocytes, K/mL, mean (SD)	4.27 (+ 3.04)	2.09 (+ 1.36)	2.40 (+ 2.38)	**0.007**†
NLR, mean (SD)	4.7 (6.7)	7.8 (10.4)	15.2 (11.5)	**0.01**‡
C-reactive protein, mean (SD)	12.56 (+ 6.79)	10.67 (+ 10.21)	15.23 (+ 11.33)	0.385
Reduced ventricular function	4 (21)	4 (16)	7 (44)	0.12
Central nervous system involvement	4 (21)	5 (21)	3 (19)	0.983
Death	0	3 (12.5)	3 (25)	0.074
Culture positivity as major Duke criteria	7 (37)	24 (96)	16 (100)	**<0.001**†
Echocardiography as major Duke criteria	16 (84)	13 (52)	10 (62)	0.083
Immunologic involvement as minor Duke criteria	0	5 (20)	4 (25)	0.078
Culture as minor Duke criteria	11 (58)	1 (4)	0	**<0.001**†
Vegetation	10 (53)	6 (24)	8 (50)	0.1

*Values are no. (%) except as indicated. Bold indicates statistical significance (p<.05). IE, infective endocarditis; NLR, neutrophil–lymphocyte ratio. †Denotes statistical significance between *Kingella* IE and both *Streptococcus* IE and *Staphylococcus aureus* IE. ‡Denotes statistical significance between *Kingella* IE and *Streptococcus* IE. §Denotes statistical significance between *Kingella* IE and *Staphylococcus aureus* IE.

### Outcome

Complications and mortality rates are shown in Table 4. No statistically significant differences were found between case-patients with KIE and those with non-KIE. Urgent surgery <10 days after admission was more common in the KIE group but did not reach statistical significance. No deaths occurred in the KIE group, whereas the non-KIE group had an intrahospitalization death rate of 17%.

**Table 4:** Complications and mortality rates among pediatric infective endocarditis case-patients, Israel, 1994–2019*

Complication	No. (%)		p value
	*Kingella* IE	Non-*Kingella* IE	
Surgical intervention	8 (42)	15 (37)	0.958
Urgent surgical intervention†	4 (8)	4 (15)	0.07
Congestive heart failure	7 (37)	7 (17)	0.192
Valvular impairment	11 (58)	15 (37)	0.233
Central nervous system involvement	4 (21)	8 (20)	1
Intrahospital death	0 (0)	6 (17)	0.131

*IE, infective endocarditis. †<10 days after diagnosis.

## Discussion

In this study we described the distinct features of pediatric KIE in a large cohort. We found that pediatric patients with KIE have similar characteristics, enabling the suspicion of *Kingella* as a causative pathogen when patients seek care. KIE is community-acquired and occurs in children (mean age 16 months) who are experiencing hyperpyrexia and have no history of previous structural heart disease. A quarter of patients in this study had a history of oral aphthae. This finding is consistent with previous studies indicating that *Kingella* are often carried in the oropharynx of toddlers and that oral aphthae are the port of entry resulting in bacteremia (*5*,*6*).

Relative lymphocytosis was found to be significantly more prevalent in the KIE group than the non-KIE group (4.27 K leukocytes/mL vs. 2.21 K leukocytes/mL). This finding is probably because of the younger age of KIE case-patients. Children with KIE had fewer positive blood cultures and shorter duration of positive cultures. When examining the Duke criteria, we found that a minority of KIE case-patients fulfilled microbiologic major criteria compared with non-KIE case-patients. Because the infection was community-acquired in all patients with KIE and only about half had structural heart disease, in most cases only 1 culture was drawn, probably because of the low level of suspicion. This practice might explain why culture positivity as a minor criterion was far more prevalent in the KIE group than the non-KIE group (57.9% vs. 2.4%). We can therefore assume that, in most cases of KIE, the diagnosis was not clear at admission and that non-KIE pathogens require prolonged antimicrobial regimens for eradication.

A previous study in Israel suggests that the proportion of pediatric KIE cases in Israel is rising, from 4.2% of total IE cases during 1980–1991 to 14% during 1994–2019 (*24*), consistent with the findings in our study. This high proportion of KIE has not been described previously in other countries (*15*,*25*). A probable explanation is the improved detection of this fastidious bacterium, combined with the tertiary nature of our medical center. In addition, a higher prevalence of *Kingella* infection in Israel is a plausible explanation (*5*,*6*).

Data characterizing the course of disease and fatal outcomes were not very helpful in differentiating between the groups, apart from deaths noted only in the non-KIE cohort, which probably signify that most KIE case-patients were healthier before contracting IE. We discovered some similarities between the KIE group and the SaIE subgroup; however, larger numbers are needed to draw significant conclusions. The similar trends observed in these groups emphasize the high risk for major complications in KIE as observed in previous studies (*16*,*26*). For reasons unknown, KIE causes devastating damage to the valve tissue in some cases but not others. This range of severity is probably explained by the different *Kingella* strains, which cause varying clinical syndromes (*27*). Unfortunately, *K. kingae* isolates of the patients in our study were not kept in our laboratory for further genotyping. A recent study postulated that a certain major virulence factor of *K. kingae* RtxA, a toxin that belongs to the RTX (repeats in toxin) group of secreted pore-forming toxins, is found in some *K. kingae* strains and causes cellular death by pore formation (*28*). Of note, *S. aureus*–derived α-toxin, a pore-forming exotoxin, has also been implicated as a major cause of cardiac tissue damage in SaIE (*29*).

The limitations of our study include its retrospective data gathering and the relatively small cohort. We did not include cases of IE caused by coagulase-negative staphylococci and enteric gram-negative bacteria in the study. These pathogens cause only nosocomial and foreign body–associated endocarditis and occur in a distinct hospital-associated population. Including those bacteria would have biased this study by further emphasizing *Kingella* as a community-acquired cause of IE. We also excluded culture-negative cases of endocarditis because these could have included partially treated cases of *Kingella* endocarditis. An additional limitation is a selection bias of the population because our medical center is a tertiary-care center. Therefore, patients in whom endocarditis is diagnosed, patients with congenital heart disease, and patients with serious complications are referred to our center from other hospitals. Conversely, this bias is preserved in all 3 groups because most pediatric patients with IE are referred to a tertiary-care center.

In conclusion, this study shows that pediatric KIE has typical features compared with StIE and SaIE. Clinical cases of high fever in young healthy children (<36 months of age), especially boys, with or without congenital heart defects and with a recent history of oral aphthae should raise the suspicion for KIE.
